# Influence of *Onopordum platylepis* Murb. as a Vegetable Coagulant on the Production and Bioactivity of Peptides in Murcia al Vino Cheese

**DOI:** 10.3390/antiox15010101

**Published:** 2026-01-13

**Authors:** Gregorio Molina-Valero, Cindy Bande-De León, Noelia Hernández-Correas, Lucia Aquilanti, Adela Abellán, Luis Tejada

**Affiliations:** 1 Department of Food Technology and Nutrition, Faculty of Pharmacy and Nutrition, Universidad Católica San Antonio de Murcia (UCAM), Campus de los Jerónimos, 30107 Murcia, Spain; cmbande@ucam.edu (C.B.-D.L.); nhernandez7@ucam.edu (N.H.-C.); aabellan@ucam.edu (A.A.); ltejada@ucam.edu (L.T.); 2 Department of Agricultural Sciences, Food and Environmental, Marche Polytechnic University, 60131 Ancona, Italy; l.aquilanti@staff.univpm.it

**Keywords:** bioactive peptides, antioxidant activity, ACE-inhibitory activity, *Onopordum platylepis*, thistle, vegetable milk coagulant, cheesemaking

## Abstract

The bioactive potential of dairy-derived peptides has attracted increasing interest due to their capacity to exert antioxidant and antihypertensive effects. This study investigated three artisanal cheeses manufactured with animal rennet (CTRL), *Onopordum platylepis* extract (OP), or a mixture of both coagulants (AR/OP) to compare their peptide profiles and associated bioactivities. Water-soluble extracts were analyzed to identify precursors and released bioactive peptides, and in vitro assays were performed to assess antioxidant activity and angiotensin-converting enzyme (ACE) inhibition. The analysis of precursors suggested a predominance of antioxidant sequences in CTRL and ACE-inhibitory precursors in OP, with AR/OP showing intermediate values. However, direct peptide identification confirmed that the AR/OP mixture produced a wider range of peptides with antioxidant activity, while OP and AR/OP exhibited similarly high levels of ACE-inhibiting peptides. These results were consistent with in vitro assays, which confirmed AR/OP as the most active sample for antioxidant potential and OP, closely followed by AR/OP, as the strongest for ACE inhibitory activity. Overall, the integration of precursor analysis, peptide identification, and experimental validation highlights the influence of the coagulant on the generation of bioactive peptides, suggesting that the use of *Onopordum platylepis* Murb. (*O*. *platylepis*) alone or in combination with animal rennet may enhance the functional properties of cheese.

## 1. Introduction

In recent decades, interest in functional foods has grown exponentially, driven by consumer demand for products that not only meet basic nutritional requirements but also support health and prevent disease. Research on functional foods not only seeks to identify new bioactive compounds but also to optimize production technologies and elucidate their interactions within complex food matrices [1,2,3].

Among the most extensively studied bioactive components, food-derived peptides have gained particular relevance due to their structural and functional diversity. These compounds have emerged as one of the most promising fields in food science and biomedicine due to their potential to exert positive effects on a wide range of physiological functions. These peptides, which are short chains of amino acids, are mainly generated from dietary proteins through enzymatic hydrolysis, microbial fermentation, or gastrointestinal digestion [1,4]. While inactive within the structure of precursor proteins, they acquire biological activity once released, exhibiting a broad spectrum of beneficial properties [2,5].

In this context, cheese is a rich source of bioactive peptides. The bioactivity of these peptides depends on structural factors such as amino acid composition, peptide chain length, and three-dimensional conformation, which collectively determine their interactions with biological targets, including receptors, enzymes, and cell membranes [6,7].

The cheese ripening process plays a central role in the generation of bioactive peptides. During this stage, which may extend over weeks or even months, milk proteins such as caseins (αs1, αs2, β, and κ) undergo extensive proteolysis mediated by endogenous milk enzymes (e.g., plasmin), coagulant enzymes, and proteases secreted by lactic acid bacteria from starter cultures or secondary microbiota. In addition, peptidases released from lysised lactic acid bacteria further contribute to proteolysis by breaking down long-chain polypeptides into short-chain peptides, thereby enhancing the diversity and abundance of bioactive sequences. These enzymes cleave peptide bonds, releasing smaller molecules with specific biological activities [1,8]. The peptide profiles generated depend on factors such as milk type (cow, goat, sheep), the microbial strains involved, specific ripening conditions (e.g., temperature, humidity, duration), and the coagulant employed.

The latter plays a decisive role, as it determines the initial enzymatic activity responsible for cleaving casein proteins and, consequently, the profile of peptides released. Traditionally, animal rennet has been employed in cheesemaking, consisting of a mixture of proteolytic enzymes, primarily chymosin and pepsin, obtained from the stomachs of young ruminants. These enzymes are highly specific, particularly chymosin, which cleaves κ-casein at the Phe105–Met106 bond, thereby destabilizing casein micelles and promoting gel formation [9,10]. Pepsin complements this activity by hydrolyzing a broader range of peptide bonds in αs1-, αs2-, and β-caseins, thereby contributing to proteolysis during cheese ripening [9,11].

More recently, plant-derived coagulants obtained from thistle species have attracted significant interest, both for their role in preserving traditional Mediterranean cheesemaking practices and for their ability to generate unique proteolytic profiles [12,13,14]. These plants contain aspartic proteases, which act differently from chymosin in animal rennet. Plant proteases are not only effective in milk coagulation but also induce distinctive protein degradation patterns that yield specific peptides, some of which have never been identified in cheeses produced with conventional rennets [15,16].

Within the group of thistle coagulants, the genus *Onopordum*, particularly *Onopordum platylepis* (*O. platylepis*), has emerged as a promising alternative [17,18]. Studies on related species such as *Onopordum acanthium* and *Onopordum tauricum* (*O. tauricum*) have confirmed the presence of onopordosins, aspartic proteases with coagulating activity [19,20,21]. Unlike animal rennet, the enzymes from *Onopordum* do not show a strong preference for the Phe105–Met106 bond of κ-casein, and their action appears to extend more broadly to other peptide bonds, resulting in a more heterogeneous and moderate proteolytic profile [21].

In this context, the present study aimed to fill a significant knowledge gap by evaluating the influence of plant-derived coagulants obtained from *O. platylepis* flowers on the generation of bioactive peptides during cheese ripening. This work was carried out using Murcia al Vino cheese, a traditional Spanish cheese protected under the Protected Designation of Origin (PDO) scheme, which is characterized by its exclusive manufacture from Murciano–Granadina goat milk, surface immersion in red wine, and controlled ripening conditions. Beyond its cultural and gastronomic relevance, Murcia al Vino represents a highly standardized artisanal system in which technological variables are tightly regulated, making it an excellent model to evaluate the impact of alternative milk coagulants on proteolysis and peptide bioactivity. Specifically, the study assessed how the proteolytic action of *O. platylepis*, alone or in combination with animal rennet, affects the peptide profile and the resulting antioxidant and ACE-inhibitory activities in this PDO cheese. By integrating the valorization of traditional plant coagulants with a product of recognized origin and quality, this work provides new insights into the development of functional dairy foods while respecting the identity of traditional cheesemaking practices [21,22].

## 2. Materials and Methods

### 2.1. Milk Coagulants

To address the objectives of this study, three types of milk coagulants were employed:

• Animal rennet: the commercial liquid rennet, obtained from ovine breeds Churra and Castellana, was supplied by Cuajos Caporal (Valladolid, Spain). The peptide extract derived from cheeses produced with this coagulant is referred to as CTRL;
• Vegetable coagulant: the plant-derived coagulant was prepared from a lyophilized extract of *Onopordum platylepis* Murb., reconstituted in chloride-free mineral water. The peptide extract obtained from cheeses produced with this coagulant is referred to as OP;
• Mixture of animal and vegetable coagulants: the peptide extract obtained from cheeses manufactured with a combination of both coagulants is designated as AR/OP.

### 2.2. Sampling of O. platylepis

Wild specimens of *O. platylepis* were collected between May and July at full flowering stage in the locality of Susa, Tunisia, Africa (Geographical coordinates: 36°31′6.738″ N, 10°56′2.078″ E). The flowers were transported to the research laboratory of the Catholic University of San Antonio de Murcia, where the styles and stigmas were separated from the remaining floral tissues.

### 2.3. Preparation of the Lyophilized Aqueous Extract

The lyophilized aqueous extracts of *O. platylepis* flowers were prepared following the procedure described by Tejada and Fernández-Salguero [23]. Briefly, styles and stigmas were macerated in distilled water at a 1:10 (*w*/*v*) ratio at room temperature in a cool, dry environment for 24 h. The resulting aqueous extract was sieved and centrifuged at 3000× *g* for 5 min. The supernatant was then filtered through Whatman No. 1 paper and stored at −32 °C for 24 h. After freezing, samples were lyophilized using a Christ Alpha 1–2 LD plus system (Osterode am Harz, Germany) under a working pressure of 4–13 Pa. The lyophilized powder was stored at −20 °C until use.

### 2.4. Manufacture of Murcia al Vino Cheeses

The cheeses were produced in an industrial facility (Quesería Tío Rest, Caravaca de la Cruz, Murcia, Spain; https://quesosdemurcia.com/queserias/tio-rest/, accessed on 15 October 2025) strictly following the specifications established by the Regulatory Council of the PDO Queso de Murcia al Vino [24].

A total of six cheeses were manufactured for each type of coagulant using pasteurized milk from Murciano-Granadina goats. The milk was preheated to 34 °C and subsequently commercial calcium chloride solution (512–532 g/L) and lyophilized starter cultures (MO-PROQ 10, Proquiga Biotech, La Coruña, Spain), composed of *Lactococcus lactis lactis* and *Lactococcus lactis cremoris* strains, were added. The corresponding coagulant was then added, and the mixture was gently stirred to ensure homogenization.

The lyophilized crude extract of *O. platylepis* was reconstituted to a concentration of 20 mg/mL. For the OP cheese, 426 mL of this solution was added per liter of milk; for the AR/OP cheese, 50 mL of the same solution plus 1.5 mL of animal rennet were incorporated. The CTRL cheese was produced exclusively with commercial liquid animal rennet, prepared according to the supplier’s instructions (4 mL per L of milk).

Coagulation occurred within 30–60 min. The curd was then cut manually with a cheese harp into medium-sized grains, followed by alternating periods of stirring and resting for 20 min. Approximately 15% of the whey was removed by washing, and the same volume was replaced with potable water at 38 °C. The curd was transferred into perforated plastic molds and pressed automatically at 1 atm for 1 h to promote whey drainage. Cheeses were salted in brine (18% *w*/*v*) for one hour, immersed in red wine for 30 min, and then transferred to the ripening chamber. The microorganism used for fermentation during wine production was *Saccharomyces cerevisiae*.

Ripening was carried out for 30 days under controlled conditions (9–13 °C and 70–90% relative humidity). During this period, the cheeses were regularly turned, cleaned, and bathed with red wine.

### 2.5. Preparation of Peptide Extracts

Peptide extracts from each cheese sample were obtained following the procedure described by Galán et al. [25]. Briefly, 15 g of cheese was weighed and mixed with 50 mL of distilled water. The mixture was then incubated in a water bath at 40 °C for 1 h. The volume was adjusted to 100 mL, and the mixture was homogenized for 1 min. Trichloroacetic acid (TCA) was added to reach a final concentration of 12%, and the solution was allowed to stand for 30 min. Finally, the suspension was centrifuged at 1500× *g* for 5 min, filtered, and stored at −20 °C until further use. Prior to the bioactivity analyses, the peptide fraction was determined using the Kjeldahl method [26].

### 2.6. Determination of Antioxidant Activity

The antioxidant activity of the peptide extracts was determined by the 2,2-diphenyl-1-picrylhydrazyl (DPPH) radical scavenging method, following the procedure described by Yang et al. [27].

Briefly, 20 μL of sample and 180 μL of DPPH solution (0.25 mM; Sigma-Aldrich, St. Louis, MO, USA) were added to each well of a 96-well plate. The mixture was incubated in the dark at room temperature for 60 min, and the absorbance was measured at 517 nm using a SpectraMax^®^ iD5 microplate reader (Molecular Devices, San Jose, CA, USA). All measurements were performed in triplicate.

The percentage of DPPH radical scavenging activity (RSA) was calculated according to the following equation:

(1) DPPH RSA (%) = [(Abs_control_ − Abs_sample_)/Abs_control_] × 100

where:

Abs_control_ represents the absorbance of the DPPH radical in the presence of water instead of the peptide extract.

Abs_sample_ represents the absorbance of the DPPH radical in the peptide extract.

Trolox equivalent antioxidant capacities (TEAC; mmol Trolox/g peptides) of the peptide extracts were also determined. For this purpose, the percentage of antioxidant activity against the DPPH radical was plotted against the concentration of Trolox (0.1–1 mM), and the linear regression equation obtained (y = 79.872x + 2.2911, R^2^ = 0.9956) was used for the conversion.

### 2.7. Determination of ACE-I Inhibitory Activity

The angiotensin-converting enzyme I (ACE-I) inhibitory activity of the peptide extracts was determined according to the method of Sentandreu and Toldrá [28], with slight modifications. This assay is based on the use of the fluorogenic peptide Abz-Gly-Phe(NO_2_)-Pro. ACE hydrolysis of this substrate generates the fluorescent product Abz-Gly, which is then quantified by fluorometry.

Briefly, 50 μL of each sample dilution and 50 μL of ACE solution (7.5 μg/mL; Sigma-Aldrich, St. Louis, MO, USA) prepared in 150 mM Tris buffer (pH 8.3; Sigma-Aldrich) were added to a 96-well plate. Subsequently, 200 μL of the substrate solution (0.45 mM o-aminobenzoylglycyl-p-nitro-L-phenylalanyl-L-proline; Sigma-Aldrich) prepared in 150 mM Tris buffer (pH 8.3) containing 1.125 M NaCl (Labkem, Barcelona, Spain) was added to each well.

Fluorescence was recorded at the time of substrate addition and after 60 min of incubation using the microplate reader mentioned in Section 2.6, with excitation and emission wavelengths of 375 and 430 nm, respectively.

The ACE-I inhibitory activity (%) was calculated using the following equation:

(2) ACE inhibitory activity (%) = [((FC_60_ − FC_0_) − (FS_60_ − FS_0_))/(FC_60_ − FC_0_)] × 100

where:

FC_60_: The fluorescence emitted by the control after 60 min.

FC_0_: The fluorescence emitted by the control at the beginning.

FS_60_: The fluorescence emitted by the sample after 60 min.

FS_0_: The fluorescence emitted by the sample at the beginning.

The analysis was performed at different concentrations for all samples. A trend line was generated, and the 50% inhibition index (IC_50_) was calculated by linear interpolation.

### 2.8. Peptide Identification

Peptide identification was performed by nano-UHPLC coupled to tandem mass spectrometry (nanoLC-MS/MS) on the peptide extracts obtained in Section 2.5, following the procedure described by Bueno-Gavilá et al. [29], with minor adaptations. Analyses were performed at the Proteomics and Bioinformatics Unit of the University of Córdoba, Spain.

Peptides were separated using a nanoElute nanoflow UHPLC system (Bruker Daltonics, Billerica, MA, USA) coupled to a timsTOF Pro 2 mass spectrometer equipped with a CaptiveSpray nano-ESI source (Bruker Daltonics). Samples were loaded onto a Bruker FIFTEEN C18 column (15 cm × 75 µm, 1.9 µm, 120 Å) and eluted at 30 °C with a linear gradient from 0–35% acetonitrile in 0.1% formic acid over 13 min, followed by a step to 90% acetonitrile, at a flow rate of 300 nL/min.

The mass spectrometer operated in positive ion mode with trapped-ion mobility spectrometry (TIMS) in data-dependent PASEF acquisition. MS and MS/MS spectra were acquired over m/z 100–1700. A collision-energy ramp of 27–45 eV was applied, and four PASEF MS/MS scans were acquired per cycle.

Raw data were processed in PEAKS Studio ProX (Bioinformatics Solutions Inc., Waterloo, ON, Canada) against the UniProtKB database. Search parameters were: precursor tolerance 15 ppm, fragment tolerance 0.05 Da, carbamidomethylation (Cys) as fixed modification, and oxidation (Met) and N-terminal acetylation as variable modifications. Peptide identification and quantification were based on peptide-spectral matches (PSMs), and quantitative values were normalized to total PSM per sample for comparison among extracts.

In addition, all identified peptides were searched in the BIOPEP-UWM database [30]. Two approaches were applied: detection of peptides with known bioactivity and identification of sequences containing fragments with potential bioactive motifs (precursors).

Data processing and statistical analyses were performed using R software (version 3.4.1).

### 2.9. Statistical Analysis

Six cheeses were produced for each treatment, representing the biological replicates. Each cheese was analyzed in triplicate, and the resulting technical replicates were averaged to obtain one value per cheese. These six averaged values (n = 6) were then used for the statistical analyses, and data are presented as mean values ± standard error. Statistical analyses were conducted using SPSS software (version 21.0; IBM Corporation, Armonk, NY, USA). Differences among samples were evaluated using one-way analysis of variance (ANOVA) for antioxidant and ACE-inhibitory activities. When significant differences were detected (*p* < 0.05), Tukey’s HSD post hoc test was used for pairwise comparisons.

Graphical representations showed in Section 3.2.2 were generated using R (version 4.3.2; R Foundation for Statistical Computing, Vienna, Austria). Hierarchical clustering was performed using Euclidean distance and complete linkage, and figures were used for the descriptive and exploratory visualization of bioactive activity profiles.

## 3. Results and Discussion

### 3.1. Antioxidant and ACE-Inhibitory In Vitro Activity

**Figure 1:**
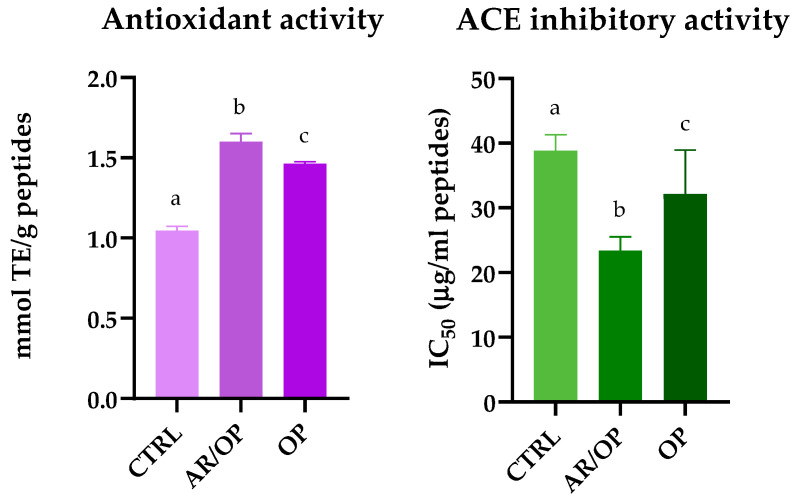
Antioxidant activity (mmol TE/g peptides) and ACE-inhibitory activity (IC50, μg/mL peptides) of peptide extracts. Different letters (a–c) indicate significant differences among treatments (*p* < 0.05; one-way ANOVA followed by Tukey’s HSD test).

Figure 1 shows the antioxidant activity values obtained for each peptide extract, expressed as millimoles of Trolox equivalents per gram of peptides. It also shows the half-maximal inhibitory concentration (IC_50_) values of ACE-inhibitory activity obtained for each peptide extract, expressed as µg of peptides per mL.

As shown in Figure 1, both the antioxidant and ACE-inhibitory activities of the AR/OP (1.60 ± 0.05 and 23.39 ± 2.13, respectively) peptide extract were higher than those of the OP (1.46 ± 0.01 and 28.22 ± 0.37) extract, with the CTRL (1.05 ± 0.03 and 38.83 ± 2.47) extract exhibiting the lowest activities. These results first highlight the remarkable ability of the combined use of plant- and animal-derived coagulants to generate bioactive peptides. This is most likely attributable to the enzymatic heterogeneity during cheese ripening, which promotes the release of unique bioactive sequences. Although the proteolytic capacity of the aqueous extract of *O. platylepis* has received little attention to date, the specificity of enzymes from other thistle extracts in the hydrolysis of milk proteins has been reported. For example, Brutti et al. [21] observed, through electrophoretic analysis, distinct proteolytic profiles between the chymosin derived from animal rennet and the onopordosins present in aqueous extracts of *O. tauricum*. Similarly, Macedo et al. [31] reported that the proteases of *Cynara cardunculus* (*C*. *cardunculus*) exhibit a clear preference for cleavage at hydrophobic regions of both αs1-casein (f163–167) and β-casein (f189–193), which were less susceptible to chymosin under various experimental conditions. Sousa and Malcata also reported differences in peptide profiles throughout all stages of cheese ripening when comparing cheeses manufactured with *C*. *cardunculus* extract and those produced with animal rennet, differences attributable to their distinct enzymatic specificities [11].

Furthermore, both the antioxidant and ACE-inhibitory activities of the peptide extracts evaluated in this study are noteworthy when compared with those reported in previous investigations. Indeed, studies have described antioxidant activity values for peptide extracts ranging from 10.9 to 167.2 μmol TE/g of peptides [32]. Likewise, IC_50_ values reported for the ACE-inhibitory activity of peptide extracts have ranged from 0.102 to 2.63 mg/mL, all of which are lower than the bioactivity levels observed in the extracts analyzed in the present work [8,33,34].

Overall, a direct comparison among the three cheesemaking conditions revealed a clear functional differentiation driven by the type of coagulant employed. Both antioxidant and ACE-inhibitory activities were significantly higher in cheeses produced with *O. platylepis* extract (OP) than in the CTRL samples. However, the highest values for both biological activities were consistently observed in the AR/OP cheeses, indicating that the combined use of animal rennet and this plant extract promotes complementary enzymatic actions that enhance peptide bioactivity beyond the effect achieved by either coagulant alone.

### 3.2. Precursor Identification

#### 3.2.1. Peptide Length and Molecular Weight Distribution

**Figure 2:**
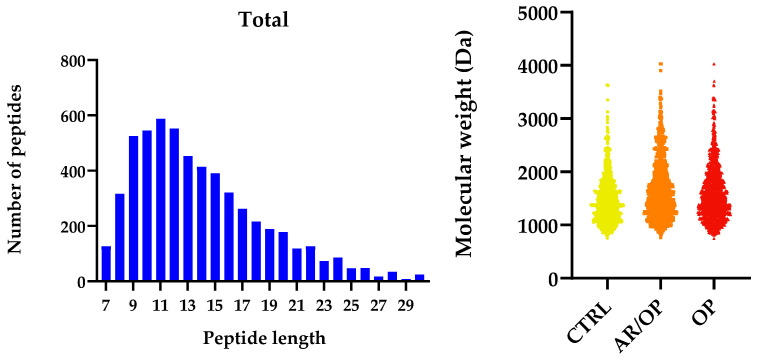
Length and molecular weight distribution of the identified peptides.

As illustrated in Figure 2, the most prevalent peptide length among all samples analyzed in this study ranged between 9 and 12 amino acid residues. At the same time, the most frequently observed molecular weights were distributed within the 1000 to 1500 Da interval.

The available evidence suggests that peptide length and molecular weight are key determinants in the diversity of bioactivities observed in food-derived peptides. Although very short fragments such as di- and tripeptides have been described as having advantages in terms of absorption and enzymatic recognition, numerous studies have demonstrated that larger peptides, with intermediate molecular masses near 1000–1500 Da and lengths up to 12 amino acids, maintain high functional capacity as long as they preserve appropriate structural motifs. This explains why potent antihypertensive effects are also observed through the inhibition of angiotensin-converting enzyme (ACE), an activity that requires specific interactions with catalytic sites in which terminal hydrophobicity and the orientation of aromatic amino acids also play a determinant role [35,36,37,38]. Likewise, medium-sized peptides have shown immunomodulatory, antiviral, and anti-inflammatory properties, consistent with studies indicating that their additional structural complexity can favor multifunctional interactions with cell receptors or signaling molecules [2,39,40]. In the antioxidant and antimicrobial fields, moderate length allows for the coexistence of aromatic and sulfur-containing residues in terminal positions together with small or aliphatic residues that provide flexibility, thereby enhancing both free radical neutralization and interaction with bacterial membranes [41,42]. Moreover, sequences of 8–12 amino acids with neuromodulatory and opioid activity have been described, in which the presence of an N-terminal tyrosine and internal or terminal phenylalanine remains essential for receptor recognition, but a minimum length is required to stabilize the bioactive conformation [43,44]. Finally, it has been observed that peptides of intermediate molecular weight also show broad metabolic effects, such as hypoglycemic [45], hypolipidemic activity [46], and the regulation of digestive enzymes [47] and intracellular signaling—confirming that bioactivity does not depend exclusively on sequence brevity, but on a combination of size sufficient to provide diverse functional motifs and the positioning of key amino acids in strategic terminal positions. Altogether, these findings support that the wide range of bioactivities observed in the peptides identified in this study is explained not only by their composition but also by their intermediate size. This appears to offer an optimal balance between structural stability, enzymatic accessibility, and functional versatility.

#### 3.2.2. Quantification of Bioactivities Based on Precursors

In line with the in vitro bioactivity results described above, a complementary peptidomic analysis was performed to explore the structural features potentially underlying the observed antioxidant and ACE-inhibitory activities. In this context, the identified peptide sequences were screened against the BIOPEP-UWM database to quantify bioactive motifs and precursor sequences associated with different biological functions. This precursor-based analysis provides an indicative framework to relate the functional outcomes to the proteolytic patterns generated by each coagulant.

**Figure 3:**
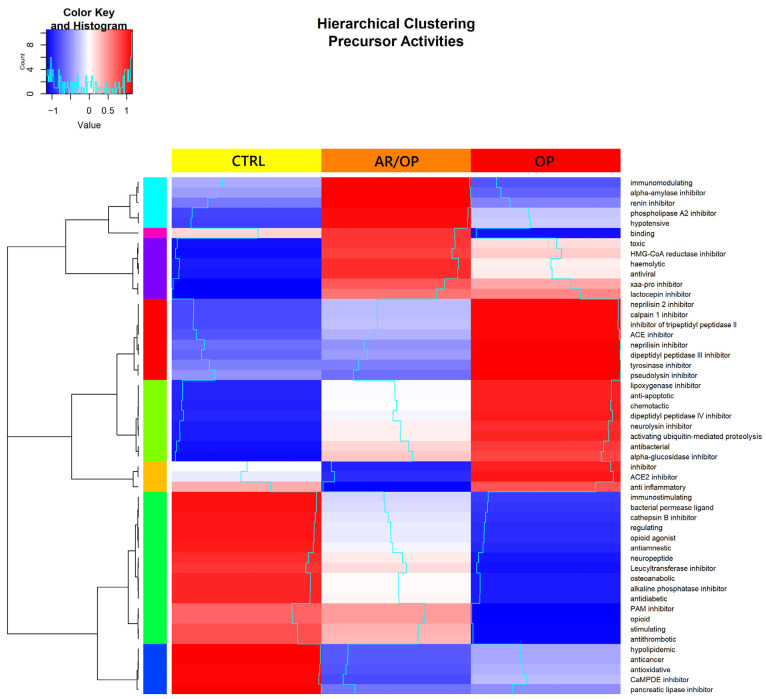
Quantitative heatmap and dendrogram of potential bioactivities inferred from identified peptide precursors in Murcia al Vino cheese samples.

As shown in the heatmap in Figure 3, the CTRL sample displayed the highest abundance of antioxidant peptide precursors the corresponding numerical values used to generate this heatmap are provided in Table S1 (Supplementary Materials). This can be ascribed to the presence of exclusive sequences and the quantitative dominance of peptide families containing histidine, aromatic and sulfur groups and hydrophobic residues, whose combined mechanisms are consistently associated with antioxidant activity in food-derived peptides. Exclusive sequences such as DQAEFKELHT and VGAHPQGLSHKVAH, which were absent in OP and AR/OP, display structural motifs coherent with these mechanisms. In DQAEFKELHT, the presence of an internal histidine provides a plausible locus for transition-metal chelation, since the imidazole side chain can coordinate Fe^2+^ or Cu^2+^ ions, thus limiting metal-catalyzed oxidative reactions. This property has been documented as one of the main pathways by which histidine-containing peptides exert antioxidant effects by stabilizing redox-active metals and mitigating radical propagation [41,48]. Beyond metal chelation, the internal phenylalanine in DQAEFKELHT may contribute to electron or hydrogen donation and the resonance stabilization of radicals, mechanisms consistently associated with aromatic residues such as Tyr, Trp, and Phe [49].

Similarly, VGAHPQGLSHKVAH, which contains three histidines and multiple hydrophobic residues (Val, Ala, Leu), combines metal-binding capacity with enhanced interfacial affinity. The hydrophobic side chains promote peptide accumulation at the water–lipid interface, thereby improving access to lipid-soluble radicals, a phenomenon repeatedly observed in food matrices [50,51].

The FPSLGEHNL-containing peptide family, which is predominantly represented in CTRL (accounting for about 75–80% of its total abundance across samples), further exemplifies this pattern. Variants such as FPSLGEHNLPHHLL, FPSLGEHNLPGAKPF, and FPSLGEHNLPKKPF share high histidine density and include aromatic and hydrophobic residues in proximity, a combination that aligns with the three canonical antioxidant mechanisms described for bioactive peptides: metal chelation, radical scavenging via electron or hydrogen transfer, and physical shielding at the water–lipid interface [42].

In addition to histidine and aromatic residues, sulfur-containing amino acids such as cysteine and methionine can also contribute to antioxidant defense. Their activity arises mainly from their ability to donate electrons or hydrogen from sulfur atoms, reacting directly with reactive oxygen species and stabilizing radical intermediates [52]. Finally, proline, frequently found in CTRL peptides, contributes to conformational stability and resistance to proteolysis, thereby prolonging peptide persistence during ripening and digestion rather than exerting direct redox reactivity [53].

Taken together, the co-occurrence of histidine-mediated metal chelation, aromatic-based electron transfer, hydrophobic-driven interfacial activity, and sulfur- or proline-mediated stability provides a comprehensive structural rationale for the enhanced antioxidant potential of CTRL. However, confirmation of functionality ultimately depends on identifying active peptides and performing direct in vitro assays.

**Figure 4:**
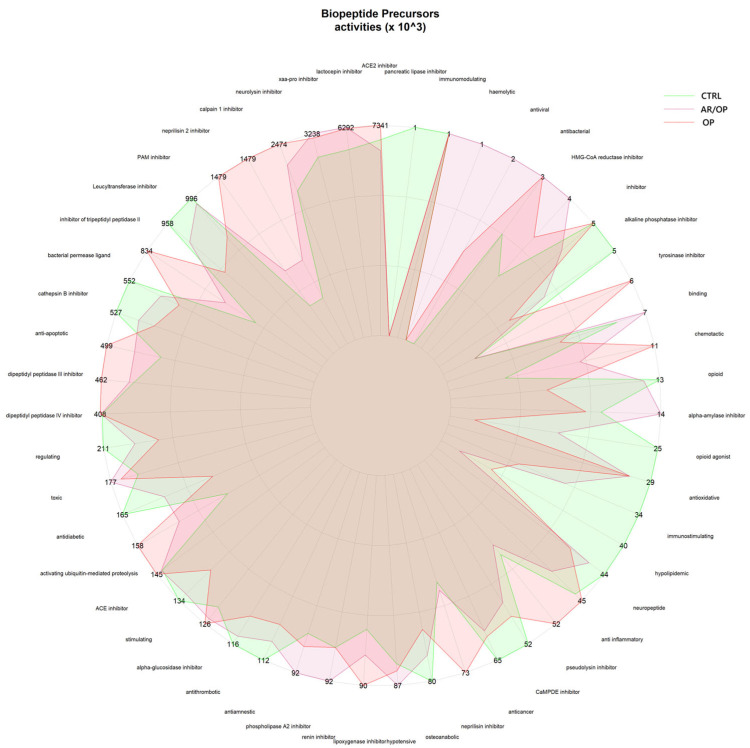
Standardized quantification of peptide precursors (×10^3^) in different cheese samples.

As also shown in the spider diagram in Figure 4, the OP sample exhibited the greatest abundance of predicted ACE-inhibitory peptide precursors. The distinctive profile of OP in the precursor analysis is consistent with a high representation of sequences bearing well-established structural traits of ACE-inhibitory peptides. In this framework, short to intermediate peptides with hydrophobic or aromatic residues near the C-terminus, often accompanied by Pro, Val or Leu motifs and occasionally basic residues (Lys and Arg), are repeatedly reported to interact favorably with the ACE binding channel [54,55,56,57,58].

Among the exclusive peptides, GVPKVKETMVPK was only identified in OP. Its C-terminal lysine provides a positive charge that can contribute to electrostatic interactions at the active site. At the same time, the presence of Val and Pro within the sequence supports hydrophobic complementarity with ACE subsites. These patterns have been repeatedly highlighted by QSAR and SAR studies [59].

Another exclusive peptide, RPKHPLNHEG, combines Pro, which confers conformational rigidity and resistance to proteolysis, with His, whose imidazole side chain can participate in hydrogen bonding or weak coordination with the catalytic zinc or nearby residues of ACE [60,61]. The sequence terminates in Glu, an acidic residue that may establish hydrogen-bonding contacts within the enzyme cleft [62]. Although this specific peptide has not been experimentally characterized, its motif architecture is consistent with the structural rules reported for ACE-inhibitory peptides, where Pro-rich cores enhance stability, and hydrophobic or aromatic residues at the C-terminus favor binding to the S1/S1′/S2′ subsites [59,61,63].

In addition to exclusives, ARHPHPHLSF and LTQTPVVVPPF were strongly over-represented in OP (≈70–75% of cumulative abundance across samples). ARHPHPHLSF terminates in Phe, an aromatic C-terminal residue that is repeatedly associated with enhanced anchoring in the S1/S1′ hydrophobic pockets; its multiple Pro residues can further enhance structural rigidity and resistance to digestion. These factors are frequently associated with greater functional persistence [61,64].

Similarly, LTQTPVVVPPF features a repeated Val–Pro motif and ends in Phe—an architecture that matches consensus patterns from QSAR/docking, where C-terminal aromatic or hydrophobic residues and Pro/Val-rich cores favor insertion into the ACE binding channel and interactions with S1/S1′/S2′ [59,63].

Overall, the presence of exclusive sequences such as GVPKVKETMVPK and RPKHPLNHEG, together with the over-representation of ARHPHPHLSF and LTQTPVVVPPF, supports the distinctive ACE-inhibitory potential of the OP sample. These peptides share structural motifs that are consistently associated with strong inhibitory activity, including C-terminal hydrophobic and aromatic residues that enhance anchoring to the S1, S1′, and S2′ subsites, Pro- and Val-rich segments that favor hydrophobic complementarity, and basic residues capable of reinforcing electrostatic interactions at the catalytic pocket. Such sequence patterns reflect the well-established structure–activity relationships described for food-derived ACE inhibitors. Nevertheless, these findings should be interpreted as predictive indicators of potential bioactivity, as they derive from precursor profiling rather than functional verification. Definitive confirmation depends on the detection of the corresponding active peptides and on direct in vitro inhibition assays.

From a comparative perspective, the precursor-based analysis further supports the differential impact of the coagulants on peptide bioactivity. CTRL was predominantly associated with antioxidant-related precursor sequences, whereas OP showed a clear enrichment in ACE-inhibitory motifs. Notably, the AR/OP samples consistently displayed a mixed precursor profile, integrating structural features associated with both antioxidant and antihypertensive activities. This distribution suggests that the combined use of animal rennet and *O. platylepis* extract promotes broader proteolytic pathways, increasing the diversity of bioactive sequences generated during ripening.

### 3.3. Bioactive Peptides Identification

Sequences of Bioactive Peptides Identified

**Figure 5:**
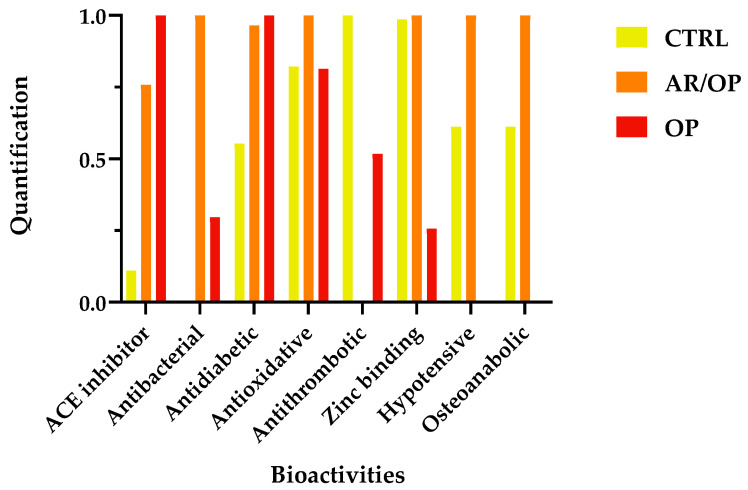
Quantification of bioactivities from bioactive peptides identified by mass spectrometry, normalized with respect to the other samples in the experiment.

Table 1 and Table 2 summarize the bioactive peptides identified in the samples analyzed in this study. Table 1 reports the molecular mass of each peptide, its protein source, and the corresponding database accession number. Table 2 presents the quantitative data for these peptides together with their reported bioactivities. Then, peptide quantification was grouped by bioactivity and normalized to 1 in order to facilitate interpretation of the results, as shown in Figure 5. The numerical values used for the normalization and graphical representation shown in Figure 5 are reported in Table S2 (Supplementary Materials).

In the present results, the AR/OP sample exhibited the highest quantification of identified antioxidant peptides. This trend not only aligns with the in vitro assays but also strengthens the overall interpretation: the presence of sequences with structural traits known to favor radical scavenging is reflected in the measured activity. Among the most abundant peptides were EDELQDKIHPF and HKEMPFPKYPVEPF. Both peptides contain aromatic residues such as Tyr and Phe, which are well-established for their ability to donate hydrogen or electrons and stabilize radicals by resonance [65,66,67]. In addition, the methionine residue in HKEMPFPKYPVEPF provides an alternative radical-neutralizing mechanism via its sulfur-containing side chain, which can react with reactive oxygen species or participate in redox cycling [52]. Histidine residues, present in several peptides, contribute to metal chelation, limiting the formation of reactive oxygen species through Fenton-type reactions by sequestering transition metals [67,68,69]. When these structural features appear together with hydrophobic residues (e.g., Val, Leu, Ile, Pro), they promote affinity for lipid environments and facilitate the scavenging of hydrophobic radicals within the cheese matrix [51]. This pattern also extends to other peptides quantified in AR/OP, such as PYVRYL, which incorporates hydrophobic and aromatic residues associated with radical-interacting capacity. Thus, the elevated abundance of these confirmed antioxidant peptides in AR/OP suggests that this processing condition promotes the accumulation of sequences structurally and functionally active in oxidative control, bridging the observed in vitro effect with their actual presence in the cheese matrix.

The quantification of ACE-inhibiting peptides was highest in the OP sample, closely followed by AR/OP, a trend that was consistent with the strong in vitro inhibition observed for both extracts. The most representative sequences—LTQTPVVVPPF, LGPRVGPFP, and TPVVVPPFLQP—share C-terminal regions enriched in hydrophobic or aromatic residues, particularly Phe or Pro. These have been repeatedly reported as key determinants of effective binding within the ACE catalytic pocket [63]. The repeated Pro residues within these peptides provide conformational rigidity and enhance resistance to proteolysis, improving both enzyme recognition and biological stability [70].

Similarly QEPVLGPVRGPFP, a β-casein-derived peptide (f194–206) previously associated with ACE-inhibitory activity [71], contains proline and a terminal hydrophobic residue arrangement (Pro–Phe–Pro) that conforms to the motif architecture favoring accommodation into the S1/S1′ subsites through hydrophobic and aromatic interactions [64]. In addition, the presence of YQKFPQY, a peptide originating from αs2-casein (f89–95) and previously reported as a bioactive sequence in dairy matrices [72], reinforces this structural pattern, as its C-terminal Tyr and internal Phe confer aromatic anchoring capacity. Moreover, Pro contributes conformational rigidity consistent with established ACE-inhibitory structure–activity relationships.

The predominance of these peptides in OP closely correlates with its superior in vitro ACE-inhibitory activity. At the same time, AR/OP’s nearly comparable levels confirm that mixed fermentation–ripening does not hinder the formation of potent inhibitory sequences.

When the identified bioactive peptides were compared across the three treatments, a consistent pattern emerged that mirrored both the in vitro results and the precursor profiling. The AR/OP samples showed the highest overall abundance of antioxidant peptides, whereas OP was particularly rich in sequences previously reported as ACE inhibitors, with AR/OP displaying comparable levels. This convergence of evidence reinforces the role of the combined coagulant strategy in shaping a bioactive peptide profile that integrates the functional strengths of both animal enzymes and onopordosins.

Taken together, the comparative evaluation of in vitro bioactivity, precursor profiling, and the direct identification of bioactive peptides provides a coherent framework to understand the influence of the different coagulants. While each analytical level highlights specific aspects of peptide bioactivity, all approaches consistently point to a differentiated behavior among CTRL, OP and AR/OP cheeses. In particular, the combined use of animal rennet and *O. platylepis* extract emerges as a distinct technological strategy that promotes enzymatic complementarity, resulting in a broader and functionally relevant peptide repertoire. This integrated interpretation establishes a clear narrative linking proteolysis, peptide composition, and biological activity.

## 4. Conclusions

The findings of this work indicate that the *O*. *platylepis* extract as a coagulant during cheese making can shape the release of bioactive peptides. Direct bioactive peptide identification showed that AR/OP contained the richest set of antioxidant sequences, whereas OP, with values very close to AR/OP, produced the most effective ACE inhibitors. The agreement between these results and the in vitro assays underlines the importance of combining predictive tools with experimental validation. Taken together, the data suggest that *O*. *platylepis* extract, particularly when used together with animal rennet, may enhance the functional properties of cheese.

## Figures and Tables

**Table 1:** Sequences of identified bioactive peptides and their protein source.

Sequence	Mass	Protein Source	Accession
LTQTPVVVPPF	1197.4188	Beta-casein OS = Capra hircus	sp|P33048|CASB_CAPHI
LGPVRGPFP	939.1089		
TPVVVPPFLQP	1193.4301		
QEPVLGPVRGPFP	1392.5971		
VVPPFLQPE	1025.1946		
EDELQDKIHPF	1370.4595		
HKEMPFPKYPVEPFTESQ	2191.4555		
HKEMPFPKYPVEPF	1746.0322		
YQKFPQY	973.079	Alpha-S2-casein OS = Capra hircus Alpha-S2-casein OS = Ovis aries	sp|P33049|CASA2_CAPHI sp|P04654|CASA2_SHEEP
PYVRYL	809.949		
TKLTEEEKNR	1247.3519		
RPKHPIKHQ	1139.6676	Alpha-S1-casein OS = Ovis aries	G3LUQ4|G3LUQ4_SHEEP
VLNENLLR	970.1212		
VPSERYL	862.9669	Alpha-S1-casein OS = Capra hircus & Ovis aries	sp|P18626|CASA1_CAPHI G3LUQ4|G3LUQ4_SHEEP
QGPIVLNPWDQVKR	1649.8859	Alpha-S2-casein OS = Capra hircus	sp|P33049|CASA2_CAPHI
HPHPHLSF	971.0698	Kappa-casein OS = Capra hircus OX = 9925 PE = 3 SV = 1 Kappa-casein OS = Capra hircus OX = 9925 GN = CSN3 PE = 1 SV = 2	A0A452G9D9|A0A452G9D9_CAPHI sp|P02670|CASK_CAPHI

Accession numbers correspond to UniProtKB protein entries (Swiss-Prot and TrEMBL).

**Table 2:** Quantification of bioactive peptides identified by mass spectrometry, expressed in arbitrary units (a.u.).

Sequence	AR/OP	OP	CTRL	Activity
RPKHPIKHQ	742	-	-	ACE inhibitor
VPSERYL	-	-	1323	
LTQTPVVVPPF	-	32,426	1254	
LGPVRGPFP	4986	7376	11,393	
TPVVVPPFLQP	4848	3860	2128	
YQKFPQY	112,713	141,649	6686	
QEPVLGPVRGPFP	8436	8742	4836	ACE inhibitor
				antidiabetic
VVPPFLQPE	-	-	8613	antioxidative
QGPIVLNPWDQVKR	407	1708	-	
EDELQDKIHPF	12,016	11,515	9081	
HKEMPFPKYPVEPFTESQ	2843	3775	3514	
PYVRYL	6474	2636	-	ACE inhibitor
				antibacterial
				antioxidative
HPHPHLSF	5721	-	3497	osteoanabolic
				antioxidative
				ACE inhibitor
				hypotensive
VLNENLLR	-	1197	-	antibacterial
TKLTEEEKNR	-	4726	9144	antithrombotic
HKEMPFPKYPVEPF	188,681	48,411	186,144	zinc binding

Quantification was performed using a label-free relative quantification approach in PEAKS Studio ProX. Peptide abundances correspond to raw ion intensities extracted from MS/MS data and are reported in arbitrary units (a.u.). Bioactivities listed in the table were not experimentally measured; these were obtained by sequence matching against the BIOPEP-UWM database, which compiles peptides with previously reported biological functions.

## Data Availability

The original contributions presented in this study are included in the article/Supplementary Material. Further inquiries can be directed to the corresponding author.

## Supplementary Materials

The following supporting information can be downloaded at: https://www.mdpi.com/article/10.3390/antiox15010101/s1, Table S1: Numerical values of the data represented in the heatmap: quantification of the precursor bioactivities (Z-scored); Table S2: Quantification of bioactivities from bioactive peptides identified by mass spectrometry, normalized with respect to the other samples in the experiment, expressed in numerical values.

## Abbreviations

The following abbreviations are used in this manuscript:

AR	Peptide extract from cheese made with animal rennet
OP	Peptide extract from cheese made with O. *platylepis* extract
AR/OP	Peptide extract from cheese made with both animal rennet and O. *platylepis* extract
O. *platylepis*	*Onopordum platylepis* Murb.
O. *tauricum*	*Onopordum tauricum*
