# Supplementary material for: Influence of Onopordum platylepis Murb. as a Vegetable Coagulant on the Production and Bioactivity of Peptides in Murcia al Vino Cheese
Source: Antioxidants (Basel). 2026 Jan 13;15(1):101. doi: 10.3390/antiox15010101 (PMC12838176; doi:10.3390/antiox15010101)
Supplement: Supplementary file 1 [file antioxidants-15-00101-s001.zip › antioxidants-4014534-supplementary.pdf]

Table S1. Numerical values of the data represented in the heatmap: quantification of the precursor bioactivities (Z-scored).

Activities	Murcia-Ani	Murcia-AO	Murcia-Ops	clusterID
ACE inhibitor	-0,773424672	-0,355824335	1,129249007	1
ACE2 inhibitor	-0,089035936	-0,952504826	1,041540762	2
activating ubiquitin-mediated proteolysis	-1,028922569	0,060601438	0,968321131	3
alkaline phosphatase inhibitor	0,98388603	0,031484353	-1,015370383	4
alpha-amylase inhibitor	-0,452338719	1,146246673	-0,693907954	5
alpha-glucosidase inhibitor	-1,101943421	0,252156512	0,849786909	3
anti-apoptotic	-0,990582071	-0,018577013	1,009159083	3
anti inflammatory	0,365006275	-1,131227446	0,766221172	2
antiamnestic	1,025934926	-0,054063174	-0,971871752	4
antibacterial	-1,082044572	0,191902453	0,890142119	3
anticancer	1,135774804	-0,748197403	-0,387577401	6
antidiabetic	0,979149402	0,040472312	-1,019621714	4
antioxidative	1,128217377	-0,777050379	-0,351166998	6
antithrombotic	0,788343644	0,33650302	-1,124846664	4
antiviral	-1,043679257	0,949683299	0,093995958	7
bacterial permease ligand	1,075525154	-0,173847432	-0,901677722	4
binding	0,1782493	0,898888705	-1,077138005	8
calpain 1 inhibitor	-0,81923895	-0,295104341	1,114343291	1
CaMPDE inhibitor	1,114315954	-0,819312446	-0,295003508	6
cathepsin B inhibitor	1,057064285	-0,126087956	-0,930976329	4
chemotactic	-1,001474552	0,002955655	0,998518896	3
dipeptidyl peptidase III inhibitor	-0,69836671	-0,447191527	1,145558238	1
dipeptidyl peptidase IV inhibitor	-0,974620066	-0,048961163	1,023581229	3
haemolytic	-1,035980138	0,959644759	0,076335379	7
HMG-CoA reductase inhibitor	-1,098886511	0,856585448	0,242301063	7
hypolipidemic	1,136122298	-0,746721701	-0,389400598	6
hypotensive	-0,85826076	1,098115694	-0,239854934	5
immunomodulating	-0,370726023	1,132422493	-0,76169647	5
immunostimulating	1,081024908	-0,189031499	-0,891993409	4
inhibitor	0,00647239	-1,003220486	0,996748095	2
inhibitor of tripeptidyl peptidase II	-0,825255594	-0,286811196	1,112066789	1
lactocepin inhibitor	-1,153447452	0,623298744	0,530148707	7
Leucyltransferase inhibitor	0,914494005	0,153306465	-1,06780047	4
lipoxygenase inhibitor	-0,988430038	-0,022751658	1,011181696	3
neprilisin 2 inhibitor	-0,81923895	-0,295104341	1,114343291	1
neprilisin inhibitor	-0,651554991	-0,499817484	1,151372475	1
neurolysin inhibitor	-1,034246253	0,07243108	0,961815173	3
neuropeptide	0,950496525	0,092569725	-1,04306625	4
opioid	0,705441575	0,43896358	-1,144405155	4

opioid agonist	1,038662645	-0,08242752	-0,956235125	4
osteoanabolic	0,98432411	0,030647218	-1,014971327	4
PAM inhibitor	0,699419042	0,445972421	-1,145391463	4
pancreatic lipase inhibitor	1,152424095	-0,638973733	-0,513450362	6
phospholipase A2 inhibitor	-0,840723945	1,105845332	-0,265121386	5
pseudolysin inhibitor	-0,490107505	-0,660399711	1,150507216	1
regulating	1,041285551	-0,088446871	-0,952838681	4
renin inhibitor	-0,599022291	1,15442589	-0,5554036	5
stimulating	0,782852945	0,343662604	-1,126515549	4
toxic	-1,071923526	0,907761395	0,164162131	7
tyrosinase inhibitor	-0,576235028	-0,578464793	1,154699821	1
xaa-pro inhibitor	-1,136443894	0,74534184	0,391102054	7

Table S2. Quantification of bioactivities from bioactive peptides identified by mass spectrometry, normalized with respect to the other samples in the experiment, expressed in numerical values.

Actividad	OP	AO	CTRL
ACE inhibitor	1	0,757681005	0,110138926
Antibacterial	0,296325254	1	0
Antidiabetic	1	0,964996568	0,553218943
Antioxidative	0,813816756	1	0,821656051
Antithrombotic	0,516880254	0	1
Binding	0,256575332	1	0,986554234
Hypotensive	0	1	0,611297946
Osteoanabolic	0	1	0,611317272
